# Nox2 underpins microvascular inflammation and vascular contributions to cognitive decline

**DOI:** 10.1177/0271678X221077766

**Published:** 2022-02-01

**Authors:** Alessio Alfieri, Juraj Koudelka, Mosi Li, Sanny Scheffer, Jessica Duncombe, Andrea Caporali, Rajesh N Kalaria, Colin Smith, Ajay M Shah, Karen Horsburgh

**Affiliations:** 1 Centre for Discovery Brain Sciences, University of Edinburgh, Edinburgh, UK; 2 National Heart and Lung Institute, Vascular Science, Imperial Centre for Translational and Experimental Medicine, Imperial College London, London, UK; 3 Department of Pathology, Amsterdam Cardiovascular Sciences, Amsterdam University Medical Centre, University of Amsterdam, Amsterdam, The Netherlands; 4 British Heart Foundation Centre for Cardiovascular Science, Queen’s Medical Research Institute, University of Edinburgh, Edinburgh, UK; 5 Neurovascular Research Group, Translational and Clinical Research Institute, Newcastle University, Newcastle-Upon-Tyne, UK; 6 Centre for Clinical Brain Sciences, University of Edinburgh, Edinburgh, UK; 7 British Heart Foundation Centre of Research Excellence, School of Cardiovascular Medicine and Sciences, King’s College London, London, UK

**Keywords:** Vascular cognitive impairment, NADPH oxidase, inflammation, cerebral hypoperfusion, white matter

## Abstract

Chronic microvascular inflammation and oxidative stress are inter-related mechanisms underpinning white matter disease and vascular cognitive impairment (VCI). A proposed mediator is nicotinamide adenine dinucleotide phosphate (NADPH) oxidase 2 (Nox2), a major source of reactive oxygen species (ROS) in the brain. To assess the role of Nox2 in VCI, we studied a tractable model with white matter pathology and cognitive impairment induced by bilateral carotid artery stenosis (BCAS). Mice with genetic deletion of Nox2 (Nox2 KO) were compared to wild-type (WT) following BCAS. Sustained BCAS over 12 weeks in WT mice induced Nox2 expression, indices of microvascular inflammation and oxidative damage, along with white matter pathology culminating in a marked cognitive impairment, which were all protected by Nox2 genetic deletion. Neurovascular coupling was impaired in WT mice post-BCAS and restored in Nox2 KO mice. Increased vascular expression of chemoattractant mediators, cell-adhesion molecules and endothelial activation factors in WT mice post-BCAS were ameliorated by Nox2 deficiency. The clinical relevance was confirmed by increased vascular Nox2 and indices of microvascular inflammation in human post-mortem subjects with cerebral vascular disease. Our results support Nox2 activity as a critical determinant of VCI, whose targeting may be of therapeutic benefit in cerebral vascular disease.

## Introduction

Vascular cognitive impairment (VCI) comprises a range of cognitive disorders caused by vascular disease and influenced by risk factors such as ageing, hypertension, diabetes and atherosclerosis.^1^ Alterations of large and small cerebral vessels, particularly those forming the subcortical white matter circulation, are key contributors to the clinical manifestation of cognitive dysfunction.^1,2^ Indeed, the extent of white matter pathology correlates with cognitive decline ^3^ and is closely related to reduced cerebral perfusion.^3–5^ Moreover, the severity of white matter alterations can predict whether patients suffering from mild cognitive impairment will develop dementia.^6,7^ VCI is common, costly and, unlike other cognitive disorders, potentially preventable and treatable,^1,2^ as the vasculature can be targeted for therapeutic intervention.

Nicotinamide adenine dinucleotide phosphate (NADPH) oxidase 2 (Nox2), a major source of reactive oxygen species (ROS) in myeloid cells and the vasculature, has been linked to several conditions of vascular pathology. Compelling evidence has related Nox2 activity to neurovascular dysfunction in ageing,^8^ angiotensin II (AngII)-induced hypertension,^9,10^ endothelin-1 (ET1)-dependent hypoxia^11^ and amyloid accumulation.^12–14^ In aged mice, neurovascular coupling measured by cerebral blood alterations is impaired by Nox2 activity.^15^ AngII-induced hypertension increases ROS vascular levels in the brain via Nox2,^9^ and impairs neurovascular coupling and cognition through Nox2 activity in perivascular macrophages.^10,16^ Furthermore, amyloid administration or over-expression in young^12^ and aged^13^ mice increase vascular ROS levels and induce neurovascular dysregulation that is ameliorated by Nox2 catalytic unit gp91 knockout. Chronic intermittent hypoxia in mice, a model relevant to obstructive sleep apnoea, also impairs cerebral blood flow (CBF) increases in the somatosensory cortex by whisker stimulation and acetylcholine, while Nox2 gp91 genetic knockout or peptide inhibitor gp91ds-tat restores these responses.^11^ There is additional evidence that Nox2 activation has a role in ageing-^17^ and disease-associated cognitive deficits.^18,19^ Oxidative damage and neuroinflammation triggered by experimental sepsis or systemic inflammation by lipopolysaccharide (LPS) are dampened by deficiency or inhibition of Nox2, thus ameliorating behavioural deficits in mice.^18,20^ Despite these links, the role of Nox2 in human VCI or vascular models of cognitive decline remains unknown.

We hypothesised that in conditions of vascular insufficiency, Nox2 activation promotes microvascular inflammation that underpins cognitive impairment, and predicted that deficiency of Nox2 would exert protective effects. To address this, we studied the effects of genetic deletion of Nox2 in a well-characterised mouse model of chronic cerebral hypoperfusion induced by bilateral carotid artery stenosis (BCAS), which develops white matter pathology, indices of vascular inflammation and spatial working memory impairments relevant to the clinical condition.^21,22^ We also tested the clinical relevance of Nox2 by measuring the expression in vessel enriched fractions from human post-mortem cases with cerebral vascular disease.

## Materials and methods

### Post-mortem human brain tissues

Human brain tissues were obtained from the Medical Research Council Edinburgh Brain Bank within the Lothian study of INtraCerebral Haemorrhage, Pathology, Imaging and Neurological Outcome (LINCHPIN) and the Lothian Birth Cohort 1936 (LBC1936).^23–26^ Control cases were obtained from sudden, unexpected, non-suspicious deaths with no known neurological disease in life. We studied the basal ganglia from cases in which small vessel disease burden was assessed as moderate or severe SVD (n = 10; 71–86 years-old; 6 male and 4 female), meeting at least the mild criteria for VCI.^27^ Control samples were gender-, ethnically- and age-matched, presenting no or only mild SVD (n = 9; 53–79 years-old; 6 male and 3 female). All samples had been collected at autopsy within 5 days from death, frozen in nitrogen vapour at –150 °C and then stored at –80 °C for further analysis. An adjacent piece of tissue from each case was fixed in 10% formalin for pathological analysis.

The Medical Research Council Edinburgh Brain Bank that has full ethical approval and consent for the use of tissue in research (East of Scotland Research Ethics Service, ref 16/ES/0084) and works within the framework of the Human Tissue (Scotland) Act 2006. Use of tissues was reviewed and approved by the Edinburgh Sudden Brain Bank ethics committee and the Academic and Clinical Central Office for Research and Development (ACCORD) medical research ethics committee at the University of Edinburgh and National Health Service Lothian. Informed consent was obtained from all participants to the LINCHPIN and LBC1936 studies, while post-mortem consent was obtained from next of kin for all other cases (sudden deaths).

### Animals

All imported wild-type (WT) mice were purchased from Charles River (UK). Nox2 knockout (Nox2 KO) mice with a targeted disruption of the NADPH oxidase subunit gp91phox were investigated^28^ and compared to WT littermates. All mice were adult male (aged 3–6 months) on a C57BL/6J genetic background. Animals were group-housed and kept on a 12 h light/dark cycle with free access to water and food (standard diet, except during behavioural assessments). All procedures were performed in a blinded manner whenever appropriate.

All animal procedures were conducted in accordance with the UK Animal (Scientific Procedures) Act 1986 under the authority of project licence number P5DE6DCE9, with local ethical and veterinary approval at the University of Edinburgh. The ARRIVE 2.0 (Animal Research: Reporting In Vivo Experiments) guidelines,^29^ as recommended by the UK National Centre for the Replacement, Refinement and Reduction of Animals in Research (NC3Rs) were followed.

### Bilateral carotid artery stenosis model

Bilateral common carotid artery stenosis (BCAS) was performed using microcoils (0.18 mm internal diameter; Sawane Spring, Japan), as previously described.^21,22,30^ Briefly, mice were anaesthetised with isoflurane (1–2% in oxygen) with the temperature maintained at 37 °C. Both common carotid arteries were exposed through a midline incision and coils placed permanently around vessels. Sham-control mice underwent the same surgical procedure without the placement of coils. Cohort 1: WT mice were coded and allocated randomly to hypoperfusion surgery: Sham (N = 13), BCAS-3d (N = 6), BCAS-6w (N = 6), BCAS-12w (N = 12). Cohort 2: mice were coded and allocated randomly to experimental groups: WT sham (WT-Sham; N =9), WT hypoperfused (WT-BCAS; N = 9), KO hypoperfused (KO-BCAS; N = 7). One mouse (WT-BCAS) tolerated surgery poorly and had to be culled. No mortality was caused by Nox2 knockout before or after BCAS surgery. Experimenters were blind to genotype and surgery status of the mice throughout data collection and analysis.

### Cerebral blood flow (CBF) and neurovascular function measurements

Laser-speckle contrast imaging was used to measure cortical cerebral perfusion at baseline and then 24 h, 6w or 12w post-BCAS surgery, using a Moor FLPI2 laser speckle contrast imager (Moor Instruments, UK). Animals were anaesthetised using isoflurane and restrained on a stereotactic frame. Body temperature was monitored throughout and maintained at 37 ± 0.5 °C using a heat pad. The skull was exposed by a midline incision and reflection of the skin of the head. The exposed skull was covered with a water-based gel and cortical perfusion measures recorded. Following recording, the skin was sutured, and a local anaesthetic applied. Animals were recovered in a temperature-regulated box prior to return to home cage. Stable blood flow recordings in the barrel cortex for 2 min were used for analysis. Speckle contrast images were analysed using MoorFLPI-2 Review software (version 4.0).

Neurovascular coupling in response to neural activity was assessed 12w after surgery under terminal anaesthesia as we previously described.^15^ In brief, mice were anaesthetised using a combination of alpha-chloralose (50 mg/kg), and urethane (750 mg/kg) injected intraperitoneally. Body temperature was monitored throughout the experiment using a rectal probe and maintained within the range of 36.5–37.5 °C using a heat blanket. Mice were placed in a stereotaxic frame and ventilated via a nose cone with 100% oxygen at a rate of 150 breaths per minute. The left whiskers were cut to minimize extraneous stimulation and the right whiskers, to be stimulated, trimmed to 1 cm. The head was fixed in place using ear and tooth bars and an incision was made over the midline. The scalp was retracted, the skull was cleaned and a thin layer of water-based gel applied to prevent the skull drying. Stable baseline blood flow in the barrel cortex was recorded for 2 min using a laser speckle contrast imager as above. The whiskers were then deflected back and forth for 30 s to stimulate blood flow to the barrel cortex. Blood flow was allowed to return to a stable baseline before beginning the next stimulation. Speckle contrast images were analysed using MoorFLPI-2 Review software (version 4.0). Peak response amplitude was recorded during stimulation, and this was expressed as % increase from baseline. Results were averaged from three stimulations. CBF recordings that were not stable before whisker stimulation were excluded from analysis.

### Assessment of spatial working memory

Spatial working memory was assessed as previously described,^21,22,30^ using an 8-arm radial maze over 16 days before the experimental end-point of 12 weeks. Mice were single-housed and food-restricted (up to 85% of initial body weight) throughout testing. Two pre-training trials were undertaken so that the animal could familiarise with the apparatus and the task itself. During testing, each arm was baited with a sugar pellet, then the mouse was placed on the maze’s central platform and monitored remotely using a camera and ANY-maze software package (Stoelting, UK). The mouse was allowed to make a free arm choice, with a trial ended when the mouse had retrieved all 8 pellets from every arm or 25 min had elapsed. For each trial, the number of revisiting errors and novel entries was recorded as a measurement of memory. Data per mouse on the first 4 trials of testing were averaged as an individual block, while 2 trials per block were calculated for the following 12d. Animals that explored less than 75% of the maze during 2 of the first 4 trials were excluded from analysis.

### Immunohistochemistry and histopathological analysis

Human formalin-fixed tissues were paraffin-embedded and sectioned at 4 µm for haematoxylin and eosin staining, followed by histopathological characterisation and assessment of SVD burden.^23,31^

At the experimental endpoint, mice were intra-cardially perfused with phosphate buffer saline (PBS) under 2% isoflurane anaesthesia. Brains were either: a) flash-frozen for total brain protein homogenisation or generation of vessel enriched fractions for subsequent molecular investigation; or b) fixed overnight in 4% paraformaldehyde (PFA) in PBS, then processed in paraffin blocks and cut into 6 µm coronal sections.

Paraffin-embedded sections were placed in 60°C oven for 30 minutes to de-wax. Following 2 washes in xylene, slides were re-hydrated in a series of ethanol (100%, 90% and 70%) and washed in running water. Immunostaining for myeloid marker Iba1 (Wako Chemicals, 019-19741) or myelin associated glycoprotein (MAG) (Abcam, ab89780) was carried out according to standard protocols. Endogenous peroxidase was quenched using 2% H_2_O_2_ in methanol for 30 minutes. Antigen retrieval was performed using 10 mM citric buffer (pH 6.0) at 110°C (for Iba1) and 95°C (for MAG) under pressure for 10 min. After cooling, slides were washed in PBS and incubated in blocking buffer (10% normal horse serum (Vector Laboratories, S-2000), 5% or 0.5% (for Iba1/MAG) bovine serum albumin (Sigma Aldrich, A7030-50G) in PBS for 1 h at room temperature. Sections were then incubated in primary antibody (Iba1 1:5000; MAG 1:30000 in block solution) at 4°C overnight. Following the overnight incubation, sections were washed in PBS and incubated in biotinylated anti-rabbit (for Iba1) or anti-mouse (for MAG) secondary antibody (Vector Laboratories, BA-1100) at 1:100 dilution in PBS for 1 h at room temperature. Signal amplification and visualisation were carried out using Vectastain Elite ABC Kit (Vector Laboratories, PK-6100) and DAB Substrate Kit (Vector Laboratories, SK-4100) kit. The slides were then dehydrated mounted in DPX. Immunostained sections were batch-imaged using a slide scanner (Zeiss Axio Scan.Z1) in brightfield. White matter damage in different brain regions was determined by MAG immunostaining and graded from 0 (none) to 3 (extensive). Myelin damage identified with MAG was determined as the presence of disorganised white matter fibres and myelin debris. The scale was as follows; normal (grade 0), minimal myelin debris, vacuolation, and disorganisation of fibres (grade 1), modest myelin debris, vacuolation, and disorganisation of fibres (grade 2), and extensive myelin debris, vacuolation, and disorganization of fibres (grade 3). Microglia density was assessed by measuring the number of Iba1 ^+^ microglia in a defined region (mm^2^) of interest. All measurements were carried out using ImageJ (v1.52r, NIH, Bethesda, MD, USA). The analysis was performed in a fully blinded manner with the investigator unaware of surgery and genotype.

### Generation of vessel-enriched fractions in human and mouse tissues

Brain vascular fractions were obtained as previously described.^32,33^ Hemibrains were homogenized with a Dounce tissue homogenizer in 1 ml (mouse tissues) or 3 ml (human tissues) PBS on ice, and the homogenate was centrifuged at 250 g for 10 min at 4 °C. The pellet was re-suspended in 3 ml of 17.5% Ficoll (Sigma) and centrifuged at 3200 g for 25 min twice. Final pellets containing the vessel-enriched fraction were collected in 1 ml of 1% BSA in PBS, re-centrifuged at 2000 g for 10 min and finally washed with 1 ml PBS before being stored at −80°C for further analysis.

### Measurement of protein levels and dot blot analysis

Protein concentration of brain tissue homogenates were assessed using the Pierce BCA Protein Assay Kit (Thermo Scientific) according to manufacturer’s instructions. Dot blotting was performed for semi-quantitative assessment of changes in protein levels, using a 96-well Bio-Rad Bio-Dot Microfiltration Unit (Bio-Rad Laboratories Ltd, UK). Samples were loaded at 2 μg of protein per well in PBS and loaded in triplicate. Samples were blotted into nitrocellulose membranes under vacuum, and the membrane was then removed and blocked in Odyssey blocking buffer (LI-COR, UK). Primary antibodies (mouse anti-gp91 1:500, BD Biosciences 611415; mouse anti-3NT, 1:750, Abcam ab61392) were diluted in Odyssey blocking buffer, incubated overnight with the membrane, washed and then incubated in secondary antibody solution (anti-mouse IR680 1:10000, LI-COR). Membranes were visualised using a LI-COR Odyssey FC scanner, and dot blots were analysed using Image Studio Lite software (LI-COR, UK). Signal intensity from each dot in the target protein channel was normalised to the loading control signal, and the results from triplicate values were averaged.

### Real time quantitative polymerase chain reaction (RT-qPCR) in human and mouse tissues

RNA was extracted from human and mouse tissues using a Qiagen RNeasy minikit according to the manufacturer’s instructions. Complementary DNA (cDNA) was generated using superscript III reverse transcriptase. Generated cDNA was combined with forward and reverse primers and SYBR Green (Applied Biosystems). Calculated cycle threshold (Ct) values were normalised to values obtained for the housekeeping gene 18S and the 2−ΔΔCT method was used to determine expression fold changes relative to the sham control group. Samples were excluded if housekeeper gene was detected >3ΔΔCt away from the mean.

### Statistical analysis

Values are reported as mean ± standard deviation (SD). Normality of data was tested using Shapiro-Wilk and thereafter appropriate parametric and non-parametric tests used. Data from the human brain tissue investigations or protein levels in mouse brain tissues were analysed using Mann-Whitney test. Data from the behavioural and cerebral blood flow analysis were analysed using a repeated-measures ANOVA followed by Bonferroni post-hoc test for multiple comparisons. Data from semi-quantitative scoring was analysed using either Mann-Whitney or non-parametric ANOVA (Kruskal-Wallis test) followed by Dunn’s post-hoc test for multiple comparison. All other data was analysed using one-way ANOVA followed by Newman-Keuls post-hoc test for multiple comparisons. Analysis and graphs were generated using Prism GraphPad 3.0 software or IBM SPSS Statistics software for analysis only. Differences with P < 0.05 were considered statistically significant.

## Results

### BCAS induces Nox2 expression, indices of microvascular inflammation and oxidative damage

**Figure 1.**
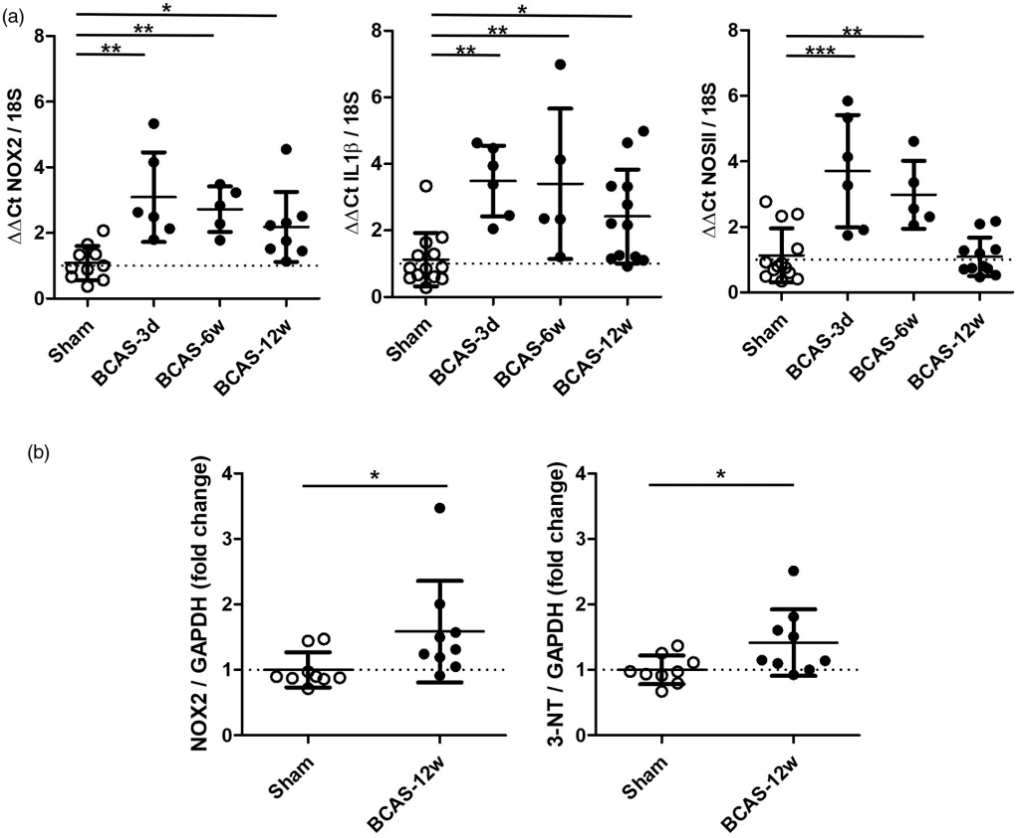
BCAS induces Nox2 expression, indices of microvascular inflammation and oxidative damage. (a) Transcription of Nox2, IL1β and NOSII in cerebrovascular fractions was significantly increased 3 days-12weeks after carotid stenosis. Statistical analysis was performed using 1-way ANOVA followed by Newman-Keuls post-hoc test for multiple comparisons. *P < 0.05, **P < 0.01, ***P < 0.001 vs. Sham; N = 4–6 per group. (b) Protein levels of 3-nitrotyrosine (3-NT) and Nox2 were increased following 12 weeks BCAS. Data was analysed using Mann-Whitney. **P = 0.0078; *P = 0.0315 vs. Sham; N = 9 per group.

The levels of Nox2 and indices of microvascular inflammation and oxidative damage were initially investigated, determining the time course of these alterations post-BCAS at 3 days, 6 weeks and 12 weeks (Figure 1(a)). Gene expression levels were measured in vessel enriched fractions isolated from wild-type (WT) mice post-BCAS and compared to sham controls. Analysis of Nox2 transcription across all groups indicated there was a significant difference (F(_3,28_) = 7.000, P = 0.0014). Post-hoc analysis indicated that Nox2 levels were significantly increased by approximately 2–3 fold post-BCAS at all time-points (3 days P < 0.01, 6 weeks P < 0.01, 12 weeks P < 0.05 vs. sham). Similarly, neuroinflammatory mediators interleukin-1β (IL1β) and inducible nitric oxide synthase (NOSII) were markedly increased post-BCAS. Initial analysis of the groups revealed a significant difference in IL1β (F(_3,32_) = 6.272, P  =  0.0018) and NOS11 (F(_3,31_) = 13.55, P < 0.0001) Post-hoc analysis showed that IL1β was elevated at all times post-BCAS (3 days P < 0.01, 6 weeks P < 0.01, 12 weeks P < 0.05 vs. sham). whilst NOSII was increased at 3 days (P < 0.01) and 6 weeks (P < 0.01) post-BCAS. Since we aimed to investigate behavioural changes at 12 weeks we sought to assess relevant protein changes at this time. Protein levels of Nox2 and 3-nitrotyrosine (3-NT), as a marker of peroxynitrate production, were determined to be significantly increased at 12 weeks post-BCAS (Nox2 P  =  0.0078; NOSII; P  =  0.0315) compared to sham mice (Figure 1(b)). Collectively, these data indicate that BCAS triggers increased Nox2 activity and microvascular inflammation in the brain, which is sustained chronically. This evidence provided a basis on which to examine the role of Nox2 in cerebrovascular injury and cognitive impairment.

### Nox2 deficiency protects against BCAS-induced cognitive impairment

**Figure 2.**
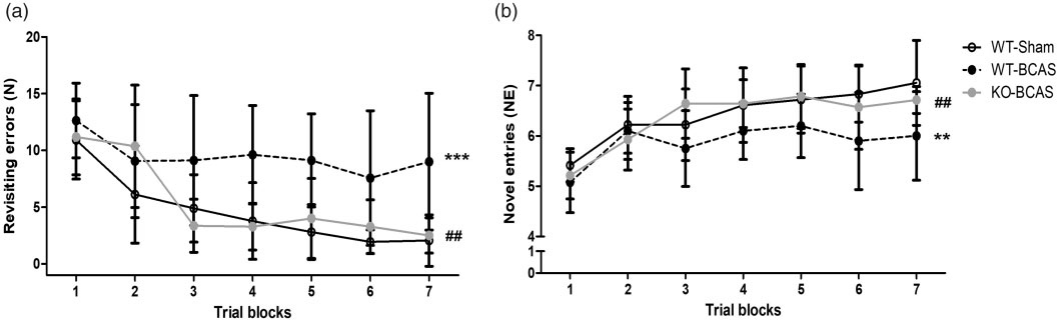
Nox2 deficiency protects against BCAS-induced cognitive impairment. Spatial working and reference memory was assessed by an 8-arm Radial Arm Maze 12 weeks post-BCAS. (a) There was a significant difference in the number of revisiting errors between WT BCAS and the sham group, while there was no difference between the Nox2 KO BCAS and sham groups. Furthermore, the Nox2 KO group showed a significantly improved performance compared to the WT BCAS group. (b) Novel entries were significantly lower in WT BCAS mice compared with sham mice, while no difference was observed between the Nox2 KO BCAS and sham groups. Furthermore, novel entries were significantly higher in Nox2 KO BCAS mice when compared with WT BCAS mice. Data was analysed with repeated-measures ANOVA followed by Bonferroni post-hoc test for multiple comparisons. ***P < 0.001, **P < 0.01 vs. WT-Sham; ^##^P < 0.01 vs. WT-BCAS. N = 7–11 per group.

We next investigated whether deficiency of Nox2 could influence the cognitive performance post-BCAS, thus studied genetically modified Nox2 null (Nox2 KO) mice compared to WT littermates. Before initiating these studies we confirmed that there was an absence of Nox2 expression in Nox2 KO mice as originally described.^28^ We and others have previously shown that BCAS causes a robust impairment in spatial working memory that can be assessed by an 8-arm Radial Arm Maze (RAM) task and measuring the number of revisiting errors and novel arm entries.^21,22,30^ In this study (Figure 2), the analysis of revisiting errors showed a significant effect of trial (F_(6,132)_ = 13.75, P < 0.0001) indicative of learning and an overall significant effect of group (F_(2,22)_ = 13.03, P < 0.001). Post-hoc analysis indicated a significant difference in the number of revisiting errors between WT BCAS and sham control groups (P < 0.001), but there was no difference between the Nox2 KO BCAS and sham control groups. Furthermore, Nox2 KO mice showed a significantly improved performance compared to WT BCAS mice (P < 0.001). Analysis of novel arm entries showed a significant effect of trial (F_(6,138)_ = 17.60, P < 0.0001) indicative of learning and an overall significant effect of group (F_(2,23)_ = 5.24, P  =  0.0133). Post-hoc analysis indicated a significant difference in the number of novel entries between WT BCAS and sham control groups (P < 0.001), but there was no difference between the Nox2 KO BCAS and sham groups. Furthermore, Nox2 KO mice showed a significantly improved performance compared to the WT BCAS mice (P < 0.001). Collectively, these findings indicate that Nox2 deficiency has a profound protective effect and restores the BCAS induced memory impairment.

### Nox2 deficiency protects against BCAS-induced white matter damage

**Figure 3.**
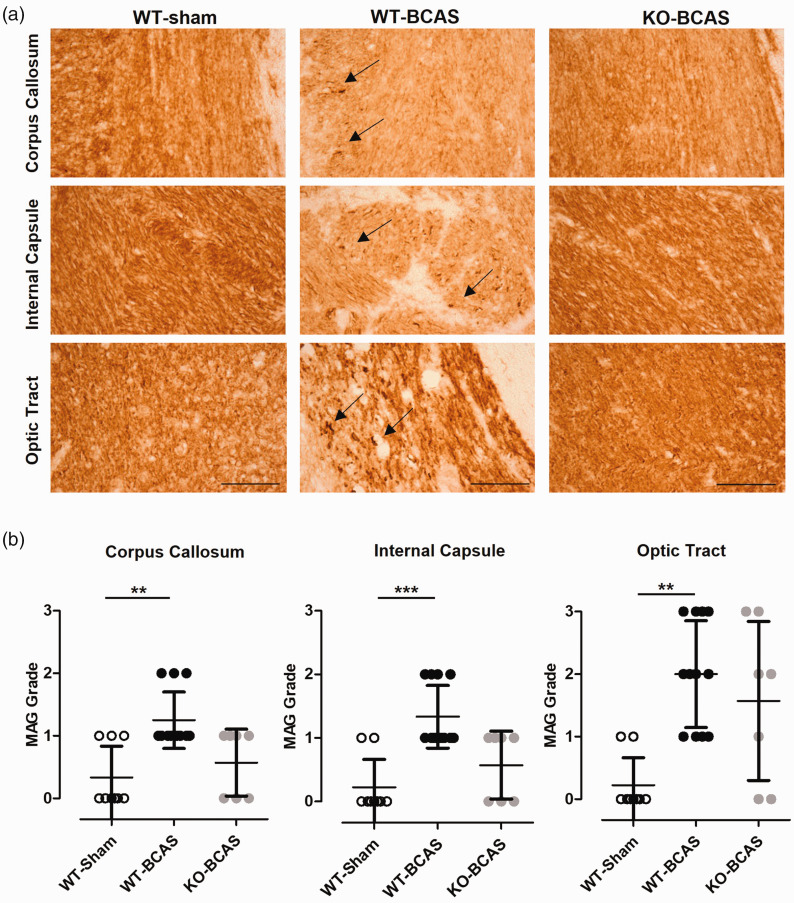
Nox2 deficiency protects against BCAS-induced white matter damage. (a) Representative images of MAG staining in the corpus callosum, the internal capsule and the optic tract from the experimental groups (WT, WT-BCAS and KO-BCAS); scale = 50 µm. Arrows point to myelin debris in WT-BCAS images. (b) White matter integrity was measured by MAG grading in the corpus callosum, the internal capsule and the optic tract at 12 weeks after surgery. MAG grading increased significantly in WT BCAS compared to shams but not in Nox2 KO BCAS mice. Data was analysed using Kruskal-Wallis test followed by Dunn’s post-hoc test for multiple comparisons. **P < 0.01, ***P < 0.01 vs. WT-Sham; N = 7–12 per groups.

The integrity of the white matter is critical for normal brain function and maintaining cognitive performances. We and others have shown previously that reduced cerebral perfusion mainly affects the integrity of myelinated axon tracts within the white matter, including the corpus callosum, the internal capsule and the optic tract.^21,30,34^ To determine if similar alterations were found in this study, the density of MAG^+^ immunostaining (as an index of axon-glial damage) was graded and the effect of Nox2 deficiency on this investigated (Figure 3). There was a significant difference in white matter integrity across the groups in the corpus callosum (H(_3_) = 12.16, P  =  0.0023), internal capsule (H(_3_) = 14.73, P  =  0.0006), and optic tract (H(_3_) = 13.25, P  =  0.0013). Post-hoc analysis revealed a significant loss of white matter integrity in WT BCAS mice compared to sham controls in the corpus callosum, (P < 0.01), internal capsule (P < 0.001) and optic tract (P < 0.01), which was not observed with Nox2 deficiency, where in all regions were not significantly different to controls. Therefore, longer-term hypoperfusion significantly induced axonal disruption in the deep white matter of WT but not of Nox2 KO mice, suggesting that cognitive deficits after chronic hypoperfusion and protective effects on cognition mediated by absence of Nox2 may be related to pathological changes in the white matter.

**Figure 4.**
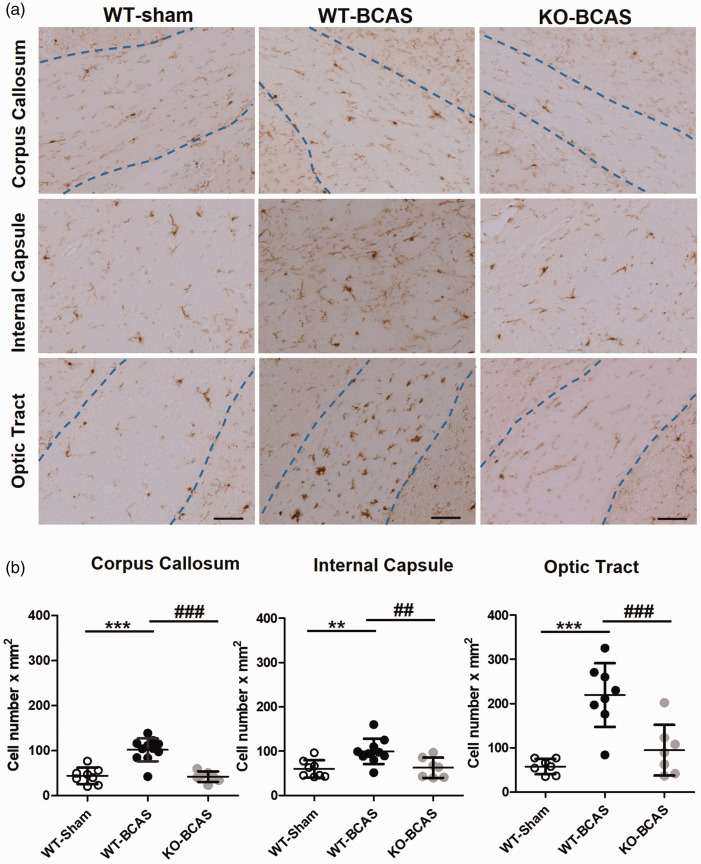
Nox2 deficiency protects against BCAS-induced microglial activation (a) Representative images of Iba1^+^ stained microglia in the corpus callosum, the internal capsule and the optic tract from the experimental groups (WT, WT-BCAS and KO-BCAS); scale = 50µm. (b) Microglial responses were analysed by Iba1 immunostaining in the corpus callosum, the internal capsule and the optic tract at 12 weeks after surgery. The number of Iba1^+^ cells was significantly higher than shams in WT BCAS mice while reverted to control levels in Nox2 KO mice. Statistical analysis was performed using 1-way ANOVA followed by Newman-Keuls post-hoc test for multiple comparisons. **P < 0.01, ***P < 0.001 vs. WT-Sham; ^##^P < 0.01, ^###^P < 0.001 vs. WT-BCAS. N = 7–12 per groups.

An impaired structural and functional integrity of the white matter over the course of chronic hypoperfusion is associated with neuroinflammation and microgliosis in the same brain regions.^21,30,35–37^ To determine whether the microglial responses in the white matter were increased in response to 12 weeks hypoperfusion and Nox2 genetic modification, Iba1^+^ staining was analysed in the corpus callosum, internal capsule and optic tract (Figure 4). At 12 weeks hypoperfusion, Iba1^+^ cell numbers were significantly different between the groups in the corpus callosum (F(_2,25_) = 24.10, P < 0.0001), internal capsule (F(_2,23_) = 7.396, P  =  0.0033) and optic tract (F(_2,19_) = 17.88, P < 0.0001). Post-hoc analysis indicated that Iba1 numbers were significantly increased in WT BCAS mice in all regions (corpus callosum (P < 0.0001), internal capsule (P < 0.01) and optic tract (P < 0.0001) compared to WT shams but were reduced significantly in Nox2 BCAS mice compared to WT BCAS in all regions (corpus callosum (P < 0.0001), internal capsule (P < 0.01) and optic tract (P < 0.0001) and were not significantly different from sham controls. Notably there was no significant alterations in Iba1^+^ cell numbers in the CA1 region of the hippocampus (F(_2,22_) = 1.287, P  =  0.2962) (Suppl Figure 1). Therefore, microglial activation may reflect the extent of white matter injury induced by chronic hypoperfusion, which was amenable to Nox2 deficiency.

### Nox2 deficiency does not affect basal cerebral blood flow but has a marked protective effect on neurovascular coupling post-BCAS

**Figure 5.**
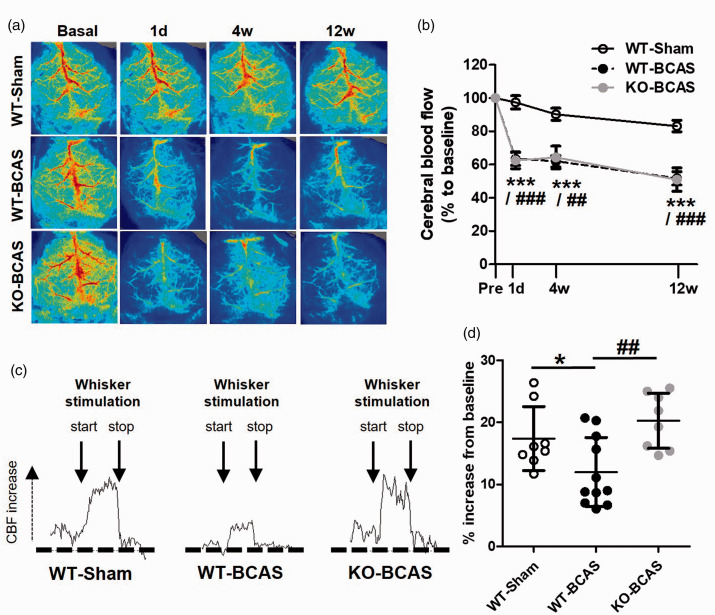
Nox2 deficiency does not affect cerebral blood flow but has a marked protective effect on neurovascular coupling post-BCAS. (a) Representative laser-speckle images from WT-Sham and WT or Nox2 KO mice undergoing BCAS at 1 day, 4 weeks and 12 weeks after surgery. (b) Cortical CBF, analysed as % change to baseline, was significantly reduced by carotid stenosis at 1 day to 12 weeks in both WT and Nox2 KO mice. Data was analysed using repeated-measures ANOVA followed by Bonferroni test for multiple comparisons. **P < 0.01, ***P < 0.001 vs. WT-Sham; ^##^P < 0.01, ^###^P < 0.001. N = 8–11 per group. (c) Representative CBF traces from laser-speckle imaging during whisker stimulation (start and stop indicated; 30 s duration) from WT-Shams and WT or Nox2 KO mice undergoing BCAS at 12 weeks after surgery. (d) Neurovascular coupling, measured as % increase in CBF during whisker stimulation at 12 weeks after surgery, was reduced by hypoperfusion in WT mice, while being increased in NOX2 KO mice. Statistical analysis was performed using 1-way ANOVA followed by Newman-Keuls post-hoc test for multiple comparisons. *P < 0.05 vs. WT-Sham, ^##^P < 0.01 vs WT-BCAS; N = 8–11 per group.

To determine if the protective effects of Nox2 could be explained by differences in CBF reductions, the extent of cortical CBF responses to BCAS was measured using laser speckle contrast imaging in Nox2 deficient and WT littermates, and compared to sham control mice. CBF was evaluated at baseline (before surgery) and then at 24 hours, 6 weeks and 12 weeks after surgery, so to assess the temporal responses to BCAS and a potential difference between genotypes (Figure 5(a)). CBF data for each animal was calculated as a percentage of its baseline CBF. Overall, there was a significant effect of time (F_(2.3,84)_ = 67.20, P < 0.001) and BCAS surgery (F_(2,22)_ = 30.99, P < 0.001) and a significant interaction between time and surgery (F_(4.60,84)_ = 7.04, P < 0.001). Post-hoc analysis indicated that CBF was significantly reduced in WT and Nox2 KO mice post-BCAS compared to sham controls at 24 hours (P < 0.001), 4 weeks (P < 0.001, P  =  0.002 respectively) and 12 weeks (P < 0.001). No effect of genotype was detected (P  =  1.00), indicating that deficiency of Nox2 does not affect cortical CBF post-BCAS.

Although CBF was not differently altered between Nox2 KO and WT mice post-BCAS, we then investigated whether the neurovascular response to neural stimulation may be changed. The coupling between neuronal activity and CBF, also known as functional hyperaemia, ensures that the metabolic needs in the brain are matched by an adequate blood flow supply. Here, neural activity was modified by whisker stimulation and blood flow changes were measured in the barrel cortex following 12 weeks BCAS (Figure 5(b); Suppl Figure 2). Overall, there was a significant effect of group on the CBF responses (F_(2,24)_ = 6.37, P  =  0.006). Post-hoc analysis showed that the CBF response was significantly blunted by BCAS (12.0 ± 1.7%) in WT mice as compared to sham controls (17.4 ± 1.8%). Moreover, the neurovascular coupling in hypoperfusion was ameliorated and restored to control levels by Nox2 deficiency (20.2 ± 1.6%; P < 0.01 vs. WT-BCAS). These results suggest that Nox2 plays an active role in mediating the altered cerebrovascular haemodynamics caused by chronic cerebral hypoperfusion.

### Nox2 deficiency reduces indices of cerebrovascular inflammation following cerebral hypoperfusion

**Figure 6.**
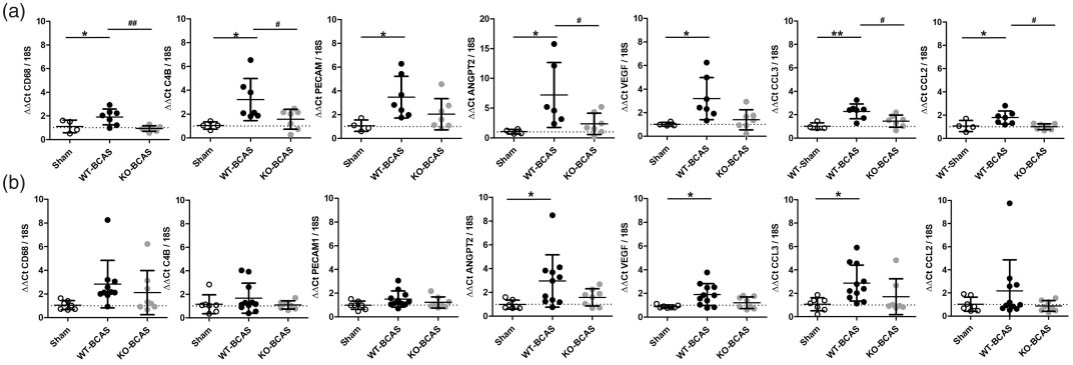
Nox2 deficiency reduces indices of cerebrovascular inflammation following cerebral hypoperfusion. Transcription of a number of markers of innate immunity, cell adhesion and endothelial activation were assessed in brain vascular fractions at 6 weeks (a) or 12 weeks (b) after BCAS surgery. Statistical analysis was performed using 1-way ANOVA followed by Newman-Keuls post-hoc test for multiple comparisons. *P < 0.05 vs. WT-Sham; ^#^P < 0.05, ^##^P < 0.01 vs. WT-BCAS. N = 6-10 per group at 6w; N = 8–12 per group at 12 weeks.

To determine if these protective effects of Nox2 deletion are linked to a beneficial impact on microvascular inflammation, we studied the transcription levels of a number of markers (innate immunity, cell adhesion and endothelial activation) in brain vascular fractions at 6 weeks or 12 weeks post-BCAS, in WT and Nox2 KO mice compared to shams. At 6 weeks, there was an overall difference in expression of CD68 (F(_2,17_) = 6.996, P  =  0.0071), C4b (F(_2,17_) = 4.901, P  =  0.023), PECAM1 (F(_2,17_) = 4.073, P  =  0.0386), ANGPT2 (F(_2,16_) = 4.751, P  =  0.0266), VEGF (F(_2,17_) = 5.250, P  =  0.0187), CCL3 (F(_2,17_) = 8.196, P  =  0.0039) and CCL2 (F(_2,17_) = 6.320, P  =  0.0102). Significant increases in the expression of these markers (CD68, C4b, PECAM1, ANGPT2, VEGF, CCL3, CCL2) were determined at 6 weeks post-BCAS (P < 0.05) (Figure 6(a)) which were restored to controls levels in Nox2 KO mice. These changes were not as prominent at 12 weeks post-BCAS (Figure 6(b)) with overall significant differences detected only in ANGPT2 (F(_2,25_) = 4.111, P  =  0.0297), VEGF (F(_2,25_) = 5.427, P  =  0.0117) and CCL3 (F(_2,25_) = 4.161, P  =  0.0293). Significant increases in the expression of these markers (ANGPT2, VEGF, CCL3) were determined at 12 weeks post-BCAS (P < 0.05) which were restored to controls levels in Nox2 KO mice. There were no overall significant differences for CD68 (F(_2,24_) = 2.327, P  =  0.1211), C4b (F(_2,25_) = 1.053, P  =  0.3651), PECAM1 (F(_2,25_) = 2.019, P  =  0.1556), and CCL2 (F(_2,25_) = 1.483, P  =  0.2478) albeit there was a trend towards an increase in expression post-BCAS and expression levels were similar in Nox2 KO post-BCAS to sham levels (Figure 6(b)). Thus collectively, our results support our prediction that Nox2 deficiency exerts beneficial effects via reductions of cerebrovascular indices of inflammation, especially chemoattractant and endothelial activation factors.

### Nox2 and indices of microvascular inflammation are increased in human small vessel disease

**Figure 7.**
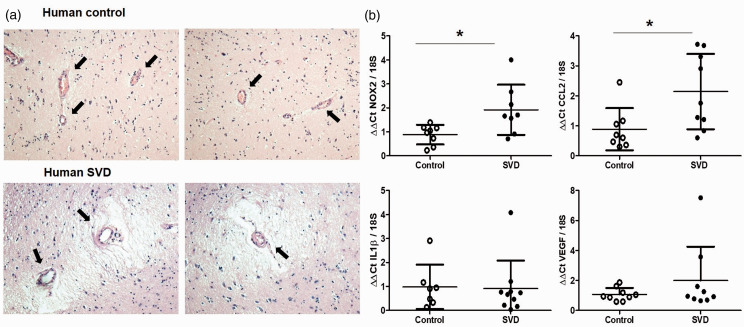
Nox2 and indices of cerebrovascular inflammation are increased in human small vessel disease. (a) Representative pictures of basal ganglia from control individuals or patients with severe small vessel disease (SVD). Histopathological analysis was used to assess the SVD burden and showed vascular pathology and microinfarcts. Images were taken at 10x magnification. (b) Transcription of Nox2 has significantly increased in vessel enriched fractions from the basal ganglia of severe SVD patients when compared to controls, together with cerebrovascular inflammation markers such as CCL2. Data were analysed using Mann-Whitney test. Nox2, *P = 0.028; CCL2 *P = 0.0161 vs. Control. N = 7–9 per cohort.

To confirm findings from our experimental model that vascular Nox2 underpins microvascular inflammation in VCI, we also investigated Nox2 expression together with indices of inflammation in cerebral vessels from post-mortem samples of SVD patients and matched controls (Figure 6). The basal ganglia of patients with high SVD burden was characterised by high vascular pathology, including arteriosclerosis, intimal thickening and microinfarcts, while both features were absent or minor in the basal ganglia from control cases (Figure 7(a)). Levels of Nox2 in vascular enriched fractions were significantly elevated (P  =  0.028) in severe SVD cases compared to control cases (Figure 7(b)). This was accompanied by significantly higher levels of immune mediator CCL2 (P  =  0.0161), while factors VEGF and IL1β are unchanged. Our analysis of human SVD cases thus confirms an increased activity of Nox2 together with evidence of vascular inflammation in human VCI.

## Discussion

Nox2 has emerged as an important redox enzyme in hypertension,^9,10,38^ Alzheimer’s disease-like amyloid pathology^13,14^ and ageing.^8,39^ Here, we demonstrate that Nox2 underpins pivotal mechanisms of cerebrovascular injury such as microvascular inflammation and neurovascular dysfunction, which ultimately contribute to white matter pathology and cognitive impairment in an experimental model relevant to VCI. Notably, vascular levels of Nox2 are confirmed as increased together with indices of cerebrovascular inflammation in human VCI.

At the outset, we firstly aimed to explore the role of Nox2 by providing a proof-of-principle investigation of its levels in the brain over the course of chronic hypoperfusion. We observed that Nox2 increased in the whole brain after 12 weeks bilateral common carotid stenosis, together with 3-NT as a marker of oxidative damage. Nox2 activity produces superoxide anion as a highly reactive species, which is then converted to hydrogen peroxide through superoxide dismutases or to peroxynitrite by direct reaction with nitric oxide.^17^ Notably, protein nitration as detected by 3-NT formation is a typical marker for oxidative damage and especially vascular oxidative injury.^40,41^ Nitric oxide is a fundamental mediator of vascular function and a reduced nitric oxide bioavailability due to its rapid conversion to peroxynitrite is consistently associated to vascular dysfunction, including in the brain.^39–43^ We also observed that Nox2 levels increased specifically in the brain vasculature, together with inflammatory mediators IL1β and NOSII. Levels of IL1β are known to be increased in stroke and chronic cerebral hypoperfusion,^44–46^ thus representing an inflammatory marker in cerebrovascular disease. Another mechanism of altered nitric oxide signalling leading to vascular dysfunction and inflammation is increased nitric oxide production through induction of nitric oxide synthases.^40^ Besides increasing the substrate for peroxynitrite formation, excessive nitric oxide leads to vasodilation, impairs vascular reactivity and promotes leukocyte recruitment. This is widely achieved in inflammation by expression of NOSII, also known as inducible NOS, the inducible form of nitric oxide synthase, which is expressed by endothelial and inflammatory cells and functions as another marker of vascular inflammation and injury.

Cognitive impairment is a defining feature of VCI, thus investigations on mechanisms and targets in VCI require the inclusion of memory assessments. Chronic cerebral hypoperfusion induced by BCAS in mice causes impairments in spatial working memory linked to disruption of frontal cortical circuitry, which supports its relevance to the clinical condition.^21,22,30,47^ Similarly, in this study, using an 8-arm RAM task we report that post-BCAS mice have impaired spatial working learning and memory. Notably, mice deficient in Nox2 post-BCAS had a marked improvement in cognitive performance compared to WT BCAS mice, and almost identical abilities to control mice. This robust protective effect of Nox2 deficiency on cognition parallels other evidence that has shown that blockade of Nox2 via pharmacological^18,19^ or genetic deletion^19^ is protective against cognitive impairments in inflammatory disease models. One notable limitation of the studies is an absence of a Nox2 control group which would have allowed the impact of Nox2 deficiency, independent of flow reductions, on behavioural and cellular outcomes.

Our pathological assessment of the white matter was focused on axon-glial disruption and cellular neuroinflammatory responses. Chronic hypoperfusion results in myelin and axonal damage that may account for deficits in white matter function.^21,30,36^ Microglial activation is an important cellular response to reduced CBF in hypoperfusion^36^ and stroke,^48^ which may contribute to axonal pathology and white matter damage by sustaining an inflammatory environment.^21,30,35–37^ We found an expected pattern of white matter damage in response to hypoperfusion that affected the optic tract, the internal capsule and the corpus callosum, and that was ameliorated by the genetic absence of Nox2. Consistent with our previous findings BCAS causes predominantly white matter pathology with minimal ischaemic neuronal damage (only one of the WT-BCAS mice had evidence of neuronal damage).^21^ Our findings collectively indicate that Nox2 mediates at least in part the hypoperfusion-induced white matter pathology and cognitive deficits, thus suggesting a potential mechanism in VCI-related white matter susceptibility and memory loss. Neuroinflammation and oxidative damage are inextricably linked, while other studies have shown an association with white matter damage induced by hypoperfusion.^30^ An association between the extent of microglial responses and white matter dysfunction was also shown previously by our group. Using electrophysiology we demonstrated an impairment in evoked compound action potentials, as an index of conduction velocity, post-BCAS which correlate with increased microglia numbers and which could be ameliorated by anti-inflammatory treatment.^36,37^

In the present study, we were able to assess changes in CBF longitudinally over the course of hypoperfusion, and neurovascular coupling at the end of experiments, via laser-speckle imaging of the brain cortex, in the same cohorts that underwent cognitive testing and assessments of white matter pathology. Furthermore, it was essential to evaluate whether Nox2 deletion per se affected basal CBF, which was not different pre-BCAS between Nox2 KO mice and WT mice. Carotid stenosis induced a significant reduction in cortical CBF by 40% ca. that was not changed by Nox2 genetic deletion. Nonetheless, the deleterious effect of sustained basal reductions in CBF is often signified by the failure of matching neural activity with blood flow supply,^34^ and we observed a prominent effect of Nox2 modification on neurovascular coupling as assessed at 12 weeks hypoperfusion. CBF increases by whisker stimulation were almost halved in response to hypoperfusion, and this effect was reverted to control levels by the absence of Nox2, an effect thus not related to basal differences in CBF between WT and Nox2 KO mice following BCAS. Neurovascular coupling is mediated by the release of vasoactive factors upon neuronal or astrocytic stimulation, such as acetylcholine, which in turn releases nitric oxide.^49^ Furthermore, oxidative damage is a detrimental mechanism mediating neurovascular dysfunction in experimental models of hypertension^10^ and amyloid pathology,^14^ by affecting nitric oxide signalling. Future studies could investigate if the CBF measures are a result of vascular and/or impaired neuronal activity. For the latter additional measures using calcium imaging or electrophysiology could be incorporated to determine if neuronal activity is impaired. Nonetheless, our findings collectively point to redox enzyme Nox2 as a major contributor to neurovascular uncoupling in response to chronic hypoperfusion, an event underlying at least in part cognitive deficits.

Mediators of innate immunity are known to be altered during cognitive decline ^50,51^ but vascular contributions to immune responses throughout VCI are unknown. In this study, we aimed to investigate a panel of immune mediators, cell adhesion molecules and endothelial activation factors specifically in the cerebral vasculature following carotid stenosis, expanding on previous investigations focused on the neuroinflammatory responses to hypoperfusion in the whole brain.^30,37^ We observed that vascular levels of immune markers CD68, C4b and cell adhesion molecule PECAM1 (CD31) were increased at 6-12weeks hypoperfusion, and reverted to control levels by Nox2 deletion. A mechanism by which myeloid cells accumulate in the brain is through cell-to-cell contacts and interaction of adhesion molecules,^52,53^ thus supporting inflammatory cell infiltration.^54^ Further investigations could be undertaken to discern potential microglia phenotype changes including phagocytic activity. Another mechanism mediating the accumulation of myeloid cells in the brain is the release of chemokines such as CCL2 (MCP1) and CCL3 (MIP1α), which function as chemoattractant factors amplifying cell recruitment in brain injury, including stroke and cerebral hypoperfusion.^55–57^ We found that CCL2 and CCL3 expression was increased in the vasculature duing hypoperfusion, and significantly reduced by Nox2 KO following carotid stenosis. Notably, both molecules are associated with the exacerbation of brain inflammation that leads to neuronal damage, while treatments reducing their levels improve outcome after injury.^37,58^ Investigation of Nox2 deficiency in specific cell-types of the brain could provide insight to Nox2 activity at a cellular level in VCI.

Endothelial cell vulnerability and dysfunction are considered critical and early events in cerebrovascular disease. Nevertheless, endothelial mechanisms leading to cognitive impairment and dementia are still primarily underestimated.^43,59^ Stroke and chronic hypoperfusion lead to endothelial oxidative damage,^60,61^ while Nox2 activity has been implicated in brain endothelial dysfunction following ischaemia.^62^ We found that mediators of endothelial activation angiopoietin-2 (Angpt2) and VEGF were significantly increased in the vasculature at 6-12weeks hypoperfusion, while Nox2 deletion reduced their expression. The Weibel-Palade bodies are specific endothelial organelles containing Angpt2 that is ready to be released upon stimulation, thus participating in platelet binding and leukocyte recruitment.^63,64^ While Angpt2 can be considered as a specific endothelial factor, VEGF is expressed by endothelial and non-endothelial cells.^43^ While activated astrocytes are the primary source of VEGF in the brain,^65^ secreted VEGF acts primarily on cerebral endothelial cells and increases blood-brain barrier permeability.^66,67^ Although it has been reported that VEGF can improve outcome after stroke and chronic hypoperfusion via its known pro-angiogenesis properties,^68,69^ these are stimulated by ischaemia upon resolution of the acute inflammatory reaction. Cognitive impairment and white matter pathology in our model are not influenced by the presence of ischaemic neuronal perikaryal damage, thus VEGF is more likely to be part of a vascular response to hypoperfusion that promotes inflammation. It is noteworthy to mention that PECAM1 and MCP1 (CCL2) have strong effects on the cerebrovascular endothelium. PECAM1 is abundantly expressed by brain endothelial cells, and its expression is increased during neuroinflammation,^70^ while MCP1 is released by glial cells and activates endothelial cells,^59^ with both mediators increasing the endothelial permeability to neutrophils and monocytes transmigration. Collectively, we showed that vascular immune responses are significantly induced by chronic cerebral hypoperfusion, suggesting that microvascular inflammation and brain endothelial dysfunction may mechanistically underlie neurovascular dysregulation and axon-glial disruption that lead to white matter damage and cognitive decline.

We finally aimed to confirm findings on the role of Nox2 from our clinically-relevant chronic hypoperfusion model in human VCI, thus analysed Nox2 levels in human post-mortem samples together with a set of immune mediators selected from our analysis in the BCAS mouse model. We selected post-mortem samples of white matter (basal ganglia) from severe SVD patients and matched controls, and performed the extraction of vascular enriched fractions for transcription analysis. Within the VCI spectrum of dementia syndromes, small vessel disease (SVD) is characterised by white matter damage with vascular pathology, likely due to microvascular alterations as a consequence to hypoxia.^1^ Notably, our model of chronic hypoperfusion recapitulates features of SVD,^22^ thus our matched analysis in the BCAS model and post-mortem SVD provides a powerful translational tool to investigate pathophysiological mechanisms in VCI. We showed that vascular Nox2 increases in severe SVD, confirming the importance of oxidative damage as a critical mechanism in human SVD,^71^ and our hypothesis on the role of Nox2 in human ageing-related brain pathology.^39^ Furthermore, we confirmed an associated increase of indices of microvascular inflammation, especially immune mediator and chemoattractant factor CCL2 (MCP1).

In conclusion, our study provides novel insights into the pathophysiology of VCI by using complementary approaches, including cognitive testing, assessments of white matter pathology and transcription analysis in both mouse and human samples. We provide evidence that Nox2-mediated oxidative damage causes CBF dysregulation and microvascular inflammation that may ultimately lead to memory impairments (Supplemental Figure 3). We suggest that these findings have important implications on the understanding of vascular contributions to dementia, and highlight the importance of mechanisms of vascular inflammation in cognitive decline.

## Supplemental Material

sj-pdf-1-jcb-10.1177_0271678X221077766 - Supplemental material for Nox2 underpins microvascular inflammation and vascular contributions to cognitive decline Click here for additional data file. Supplemental material, sj-pdf-1-jcb-10.1177_0271678X221077766 for Nox2 underpins microvascular inflammation and vascular contributions to cognitive decline by Alessio Alfieri, Juraj Koudelka, Mosi Li, Sanny Scheffer, Jessica Duncombe, Andrea Caporali, Rajesh N Kalaria, Colin Smith, Ajay M Shah and Karen Horsburgh in Journal of Cerebral Blood Flow & Metabolism
