# Supplementary material for: Nox2 underpins microvascular inflammation and vascular contributions to cognitive decline
Source: J Cereb Blood Flow Metab. 2022 Feb 1;42(7):1176–91. doi: 10.1177/0271678X221077766 (PMC9207496; doi:10.1177/0271678X221077766)
Supplement: sj-pdf-1-jcb-10.1177_0271678X221077766 - Supplemental material for Nox2 underpins microvascular inflammation and vascular contributions to cognitive decline [file sj-pdf-1-jcb-10.1177_0271678X221077766.pdf]

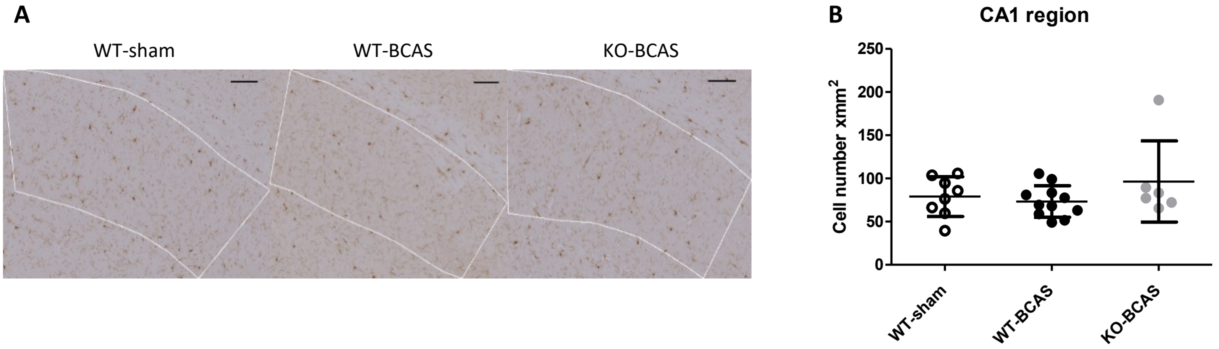

Supplemental Figure 1

(A) Representative images of Iba1 staining in the CA1 region of the hippocampus (highlighted in white) in WT-sham, WT-BCAS and NOX2 –KO, scale bar 100µm. (B) Quantification of Iba1+ cell numbers in the CA1 region of the hippocampus revealed that there were no differences overall ($F_{(2,22)}$= 1.287, P=0.2962).

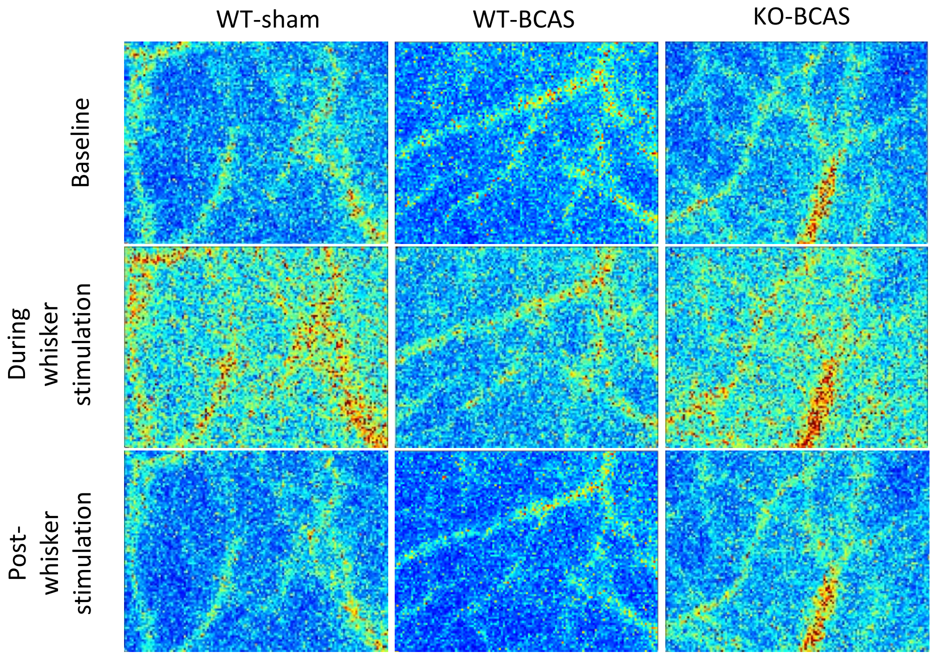

Supplemental Figure 2

Representative laser speckle images in the barrel cortex at baseline, during and post-whisker stimulation in each of the groups (WT-sham, WT, BCAS, NOX2-KO BCAS).

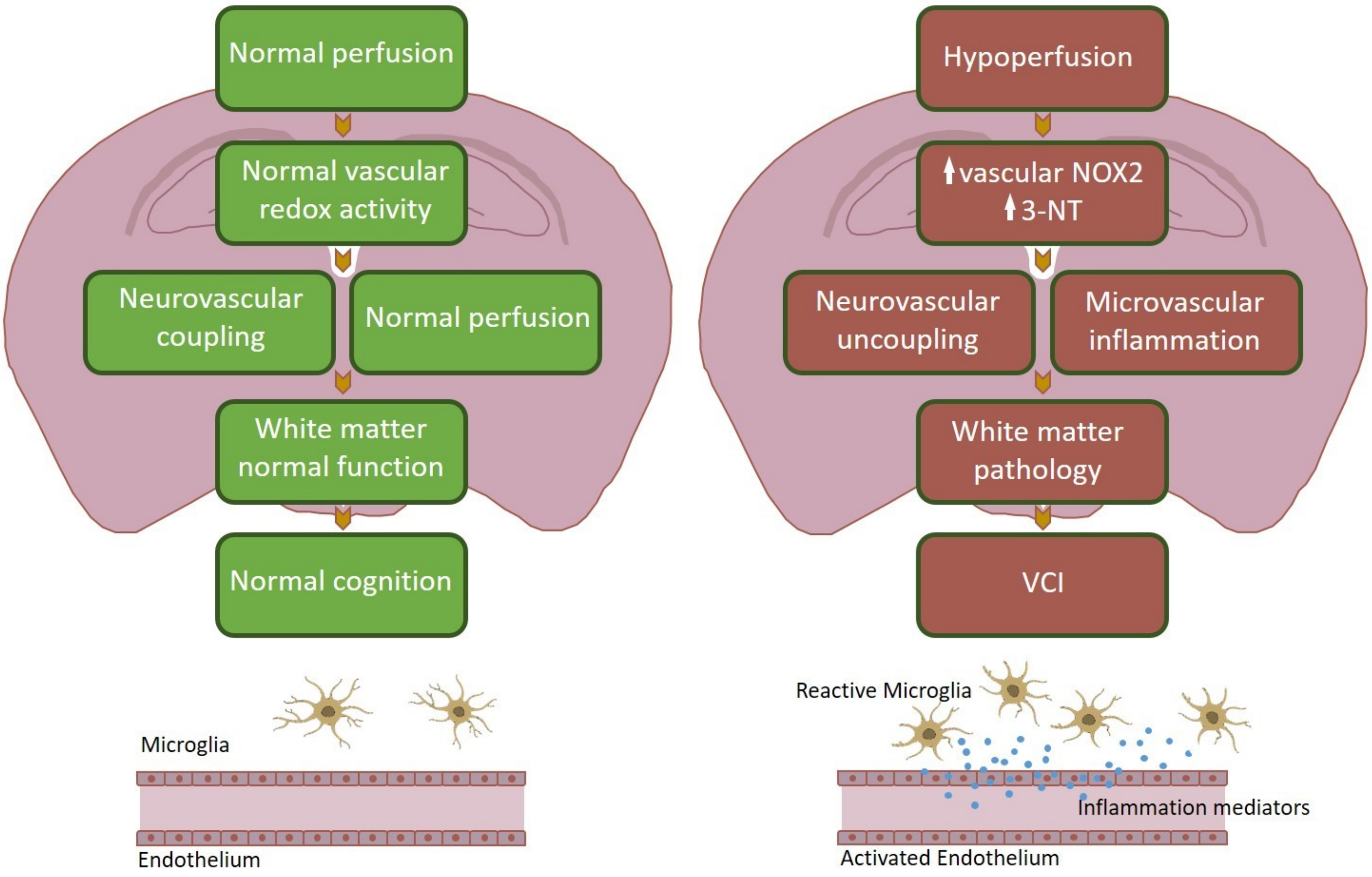

Supplemental Figure 3
Graphical summary of study
